# Proximal humeral reconstruction using nail cement spacer in primary and metastatic tumours of proximal humerus

**DOI:** 10.1007/s11751-013-0172-9

**Published:** 2013-08-08

**Authors:** Zile Singh Kundu, Paritosh Gogna, Vinay Gupta, Pradeep Kamboj, Rohit Singla, Sukhbir Singh Sangwan

**Affiliations:** Department of Orthopaedics and Rehabilitation, PGIMS, 2/11-J Medical Enclave, Rohtak, 124001 Haryana India

**Keywords:** Proximal humerus, Tumours, Limb salvage, Nail cement spacer

## Abstract

Limb salvage surgery for malignant tumours of proximal humerus is an operative challenge, where the surgeon has to preserve elbow and hand functions and retain shoulder stability with as much function as possible. We treated 14 consecutive patients with primary malignant or isolated metastasis of proximal humerus with surgical resection and reconstruction by nail cement spacer. There were 8 females and 6 males, with a mean age of 28.92 years (range 16–51 years) and a mean follow-up of 30.14 months (range 12–52 months). The diagnosis was osteosarcoma in 8 patients, chondrosarcoma in 4 patients and metastasis from thyroid and breast carcinoma in 1 patient each. One of our patients had radial nerve neuropraxia, 1 developed inferior subluxation and 3 developed distant metastasis. Two patients died of disease and one developed local recurrence leading to forequarter amputation, leaving a total of 11 patients with functional extremities for assessment at the time of final follow-up which was done using the Musculoskeletal Tumour Society (MSTS) score. Though we were able to preserve the elbow, wrist and hand functions in all patients, the abductor mechanism, deltoid muscle and axillary nerve were not salvageable in any of cases. The mean MSTS score at the time of final follow-up was 19.09. Thus, proximal humeral reconstruction using nail cement spacer is a technical simple, cost-effective and reproducible procedure which makes it a reliable option in subset of patients where the functions around the shoulder cannot be preserved despite costlier prosthesis.

## Introduction

The proximal humerus is a relatively common location for primary and metastatic tumours of bone in adults. Limb salvage surgery, instead of amputation, has become treatment of choice as it offers both functional and cosmetic advantages [1]. Various techniques have been advocated for reconstruction of skeletal defects after limb salvage. The options for reconstruction include osteoarticular allograft, allograft-prosthesis composite, free vascularized fibula graft, cement nail spacers, a sling procedure with a vascularized fibular graft, claviculo-pro-humerus and endoprosthetic replacement of the proximal humerus [1–10]. Every procedure has its own set of pros and cons, and there is no consensus on the gold standard procedure. The optimum method of reconstruction of the proximal humerus remains controversial as the function of the shoulder joint can only be restored partially as a result of various degrees of muscle loss during resection of tumour [6].

Radical removal is a principal of tumour surgery, but as much functionality as possible should be retained. These conditions often conflict, so a compromise has to be reached. When the proximal end of the humerus has to be resected, it becomes important to reconstruct it in order to give functional mobility to the upper limb. The most important issues of limb salvage surgery of proximal humerus are to maximize local control of the tumour, to preserve both elbow and hand functions and to improve shoulder stability and with as much function possible [4–8]. After resection of a malignant bone tumour of the proximal humerus, we used a nail cement spacer for limb salvage [7, 8]. The aim of this study was to evaluate the functional outcome of limb salvage surgery using nail cement spacer after wide resection of primary malignant and metastatic tumours of proximal humerus.

## Patients and methods

We retrospectively reviewed the hospital record for patients with primary malignant and metastatic tumours of the proximal humerus who were operated at our orthopaedic oncology wing between January 2005 and December 2009. There were a total of 31 patients with tumour involving the proximal humerus (metastasis *n* = 5), (primary sarcomas *n* = 26). Only those patients were included in the study in which the magnetic resonance imaging (MRI) revealed invasion of the rotator cuff or abductor mechanism with the possibility of obtaining a safe surgical margin without resecting the glenoid (Malawar classification of shoulder girdle resections type IB) [11]. Excluded from the study were patients with neurovascular bundle involvement supplying to the distal part of extremity, extensive pulmonary metastasis, soft tissue sarcomas or tumours of the clavicle, scapula or proximal part of the humeral diaphysis which did not involve the humeral head. Fourteen patients (metastasis *n* = 2), (primary sarcomas *n* = 12), fulfilled the inclusion criteria and formed the patient cohort; all these patients were managed by limb-sparing tumour excision surgery with resection of the proximal humerus and reconstruction with antibiotic bone cement [gentamycin–polymethylmethacrylate (PMMA)]-coated Kuntscher’s nail spacer. Before surgery, all patients underwent staging studies, including plain radiographs and MRI of the limb, contrast enhanced computerized tomography (CECT) scans of the chest and whole-body isotope bone scan. None of the patients had distant metastasis at the time of operation. MRI was used to define the extent of the lesion, the involvement of the soft tissues, its relation to the neurovascular bundle and the level of involvement of the bone. Preoperative histopathological diagnosis was obtained by core needle biopsy. The diagnosis was osteosarcoma in 8 patients, chondrosarcoma in 4 patients and 2 patients had single metastasis from thyroid and breast carcinoma, respectively. The primary goal of surgery was complete wide excision of the tumour, with preservation of the limb. Tumours were classified according to the Enneking’s staging system. All patients with osteosarcoma were treated with the appropriate (neo) adjuvant chemotherapy using the appropriate treatment protocols. Chondrosarcomas were treated by surgical resection only, and those with metastasis were treated with wide resection along with adequate treatment of primary and appropriate chemotherapy as per hospital protocol.

We retrospectively analysed all medical records for patient characteristics, age at diagnosis, diagnosis, surgical treatment and approach, duration of follow-up, integrity of abductor mechanism, humeral resection length measured from the tip of the greater tuberosity, resection margins, adjuvant treatment, postoperative complications, oncological parameters including overall survival, and local or systemic relapse.

The lesions were approached by way of an extended deltopectoral anterolateral incision, the exact position of which was determined by the site of biopsy and the location and extent of the tumour. Previous biopsy tracts were incorporated into the incision and were completely excised. All were transarticular resections, leaving the glenoid intact. The glenohumeral joint was disarticulated by dividing the long head of biceps as well as the tendinous portion of the rotator cuff. The tendons of pectoralis major, latissimus dorsi, teres major and the long head of biceps were detached. A cuff of normal soft tissue was retained around the proximal humerus so as to complete the ‘wide excision’. Meticulous dissection was carried out, and an intraarticular proximal humerus with the humeral diaphysis was isolated at least 2.5 cm from the most distal part of the lesion (as determined by MRI) and cut using an oscillating saw. Marrow from remaining distal humeral diaphysis was sent for frozen section evaluation.

Once the tumour was excised, haemostasis was achieved. The glenoid and remaining humerus were prepared to accept the implant. The humeral canal was reamed to accept the intramedullary nail. Depending upon the length of humerus resected, Kuntscher’s nail antibiotic cement (PMMA) spacer was prepared, moulding the semisolid cement around the nail to provide the shape and volume of resected humerus. At the proximal end, cement was moulded to provide the shape of humeral head. The distal end of nail inserted into the reamed intramedullary canal which was filled with cement for better fixation. The longest possible nail was used to construct the spacer. The cement head made at proximal end was abutted into the glenoid. Soft tissue reconstruction was completed mainly through crossed suture and reattachment of the residual muscles around the shoulder girdle to provide static stability. The residual muscles were anchored to the nail spacer with the help of four to six sutures passed through holes made in cement before setting when it was solid and mouldable, using braided non-absorbable No. 2 Ethibond suture. Soft tissue and skin were sutured over a negative suction drain. Postoperatively the arm was placed in an arm chest bandage. Stitches were removed after 3 weeks, and the hand, wrist and elbow were mobilized. After 6–8 weeks, the sling was removed and passive mobilization began. They were then followed up at regular intervals and were assessed for local control, function and complications related to the implant. Functional assessment at the time of final follow-up was done using the Musculoskeletal Tumour Society (MSTS) functional scores [12].

## Results

There were 8 females and 6 males. The mean age at the time of surgery was 28.92 years (range 16–51). The mean follow-up was 30.14 months (range 12–52 months). The mean length of resected bone was 12 cm (range 9–15). The details of the patients profile and the final outcome is given in Table 1. All resection margins were histologically free of disease on intraoperative frozen sections and final analysis. The abductor mechanism, deltoid muscle and axillary nerve were not salvageable in any of the 14 cases. There were no major intraoperative complications (neurovascular) and no superficial or deep infections. One patient had postoperative radial nerve neuropraxia, which recovered in 5 months, and another patient developed inferior subluxation of the proximal humeral head associated with a dragging sensation and paraesthesia due to shoulder instability. Ten patients remained free from disease till final follow-up. One of the patients with osteosarcoma had a local recurrence after 26 months of follow-up and underwent forequarter amputation. One patient with osteosarcoma had lung metastasis and died 16 months after surgery, and another patient with breast carcinoma had lung and brain metastasis and died 12 months after operation. A patient with chondrosarcoma was diagnosed to have a lung metastasis after 20 months of surgery. The patient is not willing for any further surgical intervention but is still under follow-up at 24 months.

**Table 1:** Demographic profile of the patients and the functional outcome

S. No.	Age/sex	Diagnosis	Pathological fracture	Stage	Complications	MSTS score	Metastasis	Latest status	Follow-up (in months)
1	28/F	Osteosarcoma	No	IIB	–	20	–	CDF	48
2	17/M	Osteosarcoma	No	IIB	–	19	–	CDF	30
3	32/F	Chondrosarcoma	No	IIB	–	23	–	CDF	52
4	16/F	Osteosarcoma	No	IIB	–	19	–	CDF	32
5	51/F	Chondrosarcoma	No	IIB	–	16	–	CDF	40
6	44/F	Metastasis from thyroid	Yes	–	–	18	–	CDF	36
7	22/M	Osteosarcoma	No	IIB	–	–	Lung	DOD	16
8	20/F	Osteosarcoma	No	IIB	Radial nerve neuropraxia	21	–	CDF	18
9	17/M	Osteosarcoma	No	IIB	Local recurrence	–	–	DF	26
10	48/M	Chondrosarcoma	No	IB	–	15	Lung	AWD	24
11	34/F	Chondrosarcoma	No	IIB	Inferior subluxation	18	–	CDF	36
12	18/M	Osteosarcoma	Yes	IIB	–	19	–	CDF	28
13	16/M	Osteosarcoma	No	IIB	–	22	–	CDF	24
14	42/F	Metastasis from breast	No	–	–	–	Lung, brain	DOD	12

*M* male, *F* female, *CDF* continuous disease free, *DF* disease free, *DOD* died of disease, *AWD* alive with disease

There was no case of cement implant loosening, implant failure or fracture. Wrist and fine movements of the hand were preserved in all patients, although elbow extension was limited in 3 cases in the early postoperative months which gradually improved to almost full extension with physiotherapy. All the patients were able to perform their day-to-day activities and routine work (hand and face washing, eating, lifting a cup and other household works). Functional data were available for 11 patients with functional extremity at the time of final follow-up (Table 1). The mean MSTS score was 19.09 (range 15–23) with the mean overall functional rating of 63.63 % (range 50–6.67 %). With regard to pain, emotional acceptance and manual dexterity, the results were rated as satisfactory with a score of 3.0 points or more in 11 patients.

## Discussion

For high-grade malignant tumours of the shoulder girdle, limb salvage surgery rather than amputation has become treatment of choice in last few decades as it offers both functional and cosmetic advantages. Limb salvage is socially and emotionally easier for patients to accept than amputation. Most replacements of the proximal humerus act as functional spacers rather than as an articulating reconstruction. The optimum method of reconstruction of the proximal humerus remains controversial as the function of the shoulder joint can only be restored partially as a result of various degrees of muscle loss during resection of tumour [6].

When evaluating a reconstruction technique, the factors which need to be considered include the ease of the procedure, its morbidity, complications, functional outcome and durability. In the past, the wide resection with no reconstruction at all was done and healing occurred by fibrosis. However, a salvaged flail shoulder may result in traction neuropathy and reduced function of the hand, forearm and elbow, due to mechanical instability [13].

Shoulder arthrodesis after resection requires graft augmentation, which is further fraught with the risk of fatigue fractures or failure of fixation [14]. The use of the avascular strut allograft is often limited by the available length of the resection, risk of non-union, fracture and infection, besides the fear of disease transmission. Vascularized fibular grafts specifically require microsurgical expertise and entail longer operating times and increased blood loss without an improved functional outcome. Further, it adds morbidity to the normal limb [4]. Free fibular graft from contralateral leg in the presence of extensive dissections, especially if it is resected for more than 12 cm, may lead to fracture and failure of incorporation of graft and, even if it survives, will take a very long time to heal with poor functional outcome [7, 15]. Endoprosthesis is the most common mode of reconstruction nowadays, but its high cost (more than 2,000 US $) is the major limiting factor in many parts of the world. Furthermore, in extra-compartmental bone tumours of the proximal humerus, the rotator cuff has to be resected (Malawer resection type IB) [11]. Shoulder function is directly related to the restoration of rotator cuff function. If this proves to be impossible, the patient ends up with an unstable joint and unsatisfactory function with compromised active positioning of the hand and poor lifting ability. With resection of deltoid muscle, rotator cuff and axillary nerve, the prosthesis replacement has to overcome failure of humeral fixation, superior head migration and lack of muscle insertion, finally acting as a passive spacer [7, 16]. Although newer techniques and prosthesis are coming up to meet the deficiencies, it further adds to the cost and requires expertise [16, 17].

**Fig. 1:**
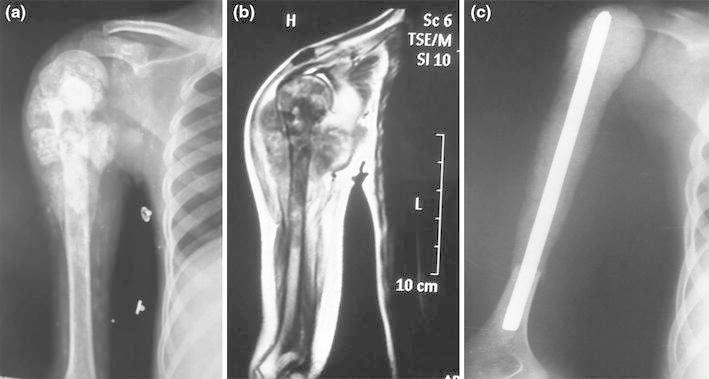
**a** Preoperative X-ray of osteosarcoma after neo-adjuvant chemotherapy. **b** MRI showing the extent in the soft tissue and in the medullary canal **c** Postoperative X-ray showing the Kuntscher’s nail cement spacer after subtotal resection of the humerus

**Fig. 2:**
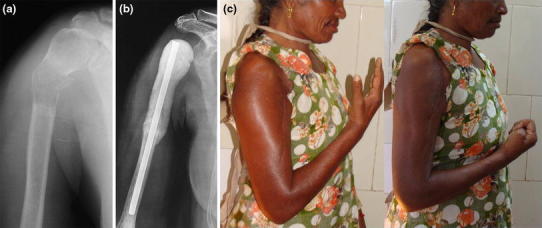
**a** Metastasis from thyroid preoperative X-ray. **b** Postoperative X-ray showing the Kuntscher’s nail cement spacer. **c** Good flexion of elbow and hand movements

The choice of reconstruction technique should be based on the extent of the resection and the need of the patient. Most authors agree that after reconstruction of extensive proximal humeral lesions, the shoulder function is compromised [6]. Stability at the proximal end of the reconstruct ensures good hand and elbow function. Although little function is restored to the shoulder, such reconstructions provide a stable fulcrum for function of the elbow and hand and prevent pain related to traction on the neurovascular bundle. Reconstructing these defects using this cement K-nail spacer is an inexpensive (the implant with cementation costs less than US $100) and effective method, which gives adequate shoulder and arm stability and ensures excellent hand and elbow function (Figs. 1, 2). Furthermore, the operative time is short, and the procedure is less technically demanding [7]. The complications in bone graft incorporation due to the use of adjuvant chemotherapy and radiotherapy leading to a delay in postoperative rehabilitation are avoided with this metallic implant–cement spacer. This method offers immediate distal fixation and early administration of radiotherapy in immediate postoperative period if required [8]. Unlike the lower limb, which is subject to variable stresses and loading, the upper limb faces relatively less intense biomechanical forces, and this could be the reason why none of our reconstructs needed revision so far. The use of antibiotic cement provides higher concentration of local antibiotic and helps in combating local infection. Extensive resections may often compound the problem if the remaining distal stump of bone is very small. This K-nail cement spacer with intramedullary nail can be used even in these cases with shorter lengths. Even endoprosthesis needs a definite amount of residual host bone for adequate fixation of the stem after resection, and this is a limiting factor to their use in such cases [11]. Reconstructing these defects using custom-made plates has been advocated, but the number of screws through the distal fragment is limited with the risk of implant failure [10].

The 14 patients who had reconstruction with a functional spacer generally fared well from a reconstructive standpoint. One of the problems related to these spacers was subluxation of spacer from glenoid fossa. Van de sande et al. [18] compared the outcomes after transarticular tumour resection and proximal humeral reconstructions using allograft-prosthesis composite (*n* = 10), osteoarticular allograft (*n* = 13) or a modular tumour prosthesis (*n* = 14) over a mean follow-up of 10 years. There was one case of subluxation in osteoarticular group, one case of dislocation in modular prosthesis group and 3 cases of subluxation and 1 case of dislocation in allograft-prosthesis composite group. Scotti et al. [19] in there series of 40 cases of proximal humeral metastasis managed with endoprosthetic reconstruction reported superior dislocation of the humeral head in 3 cases. The subluxation/dislocation rate in the present series is much lower than that reported with other procedures. This could be attributed to the use of proline mesh, which we applied around the spacer in all our cases. Ioannou et al. [20] in their study to evaluate the postoperative outcomes of reconstructive surgery for malignant and aggressive benign tumours of proximal humerus identified that that stabilization of the prosthesis with the use of mesh avoids instability. Marulanda et al. [21] also advocated the use of a synthetic vascular mesh for proximal humerus reconstruction. In their study of 16 patients with proximal humerus replacements reconstructed with a synthetic mesh, with a follow-up ranging from 13 to 43 months, there was not even a single case of shoulder dislocation. The present study also supports the evidence in favour of mesh reconstruction for proximal humerus reconstruction which reduces subluxation/dislocation and facilitates soft tissue attachment and reconstruction after tumour resection.

There was one case of neuropraxia in the current series. Bickel et al. [22] reported 13 transient nerve palsies in there series of 134 patients who underwent limb-sparing resection for tumours around shoulder girdle. Though loosening of the cemented stem of the modular spacer within the humeral canal was not seen in our series, one should be vigilant enough to look for these changes clinico-radiologically as there may be pain and resorption at the junction of the reconstruct and the humerus.

The functional, psychological, emotional and cosmetic results were acceptable to all our patients and were better than those that have been reported after amputation and use of external prostheses [13]. Furthermore, this spacer can be converted to other available options at any time. If the patient has financial constraints, expected survival time is short (metastasis) and only moderate orthopaedic oncology infrastructure is available; then, the Kuntscher’s nail and cementation method is an acceptable treatment. The final decision of the procedure is influenced by patient’s age, functional condition, stage of tumour, degree of soft tissue involvement and available expertise and experience of the surgeon. Cemented Kuntscher’s nail spacer offers a cost-effective limb salvage procedure with preservation of elbow and hand. The low cost of the implant makes it a good alternative option of treatment in these selected indications.
